# Salvaging Detection of Early-Stage Ovarian Malignancies When CA125 Is Not Informative

**DOI:** 10.3390/diagnostics11081440

**Published:** 2021-08-10

**Authors:** Charles J. Dunton, Megan L. Hutchcraft, Rowan G. Bullock, Lesley E. Northrop, Frederick R. Ueland

**Affiliations:** 1 Aspira Women’s Health, Inc., 12117 Bee Caves Road, Building III, Suite 100, Austin, TX 78738, USA; cdunton1@aspirawh.com (C.J.D.); lnorthrop@aspirawh.com (L.E.N.); 2 The Women’s Hospital, Evansville, IN 47630, USA; 3 Division of Gynecologic Oncology, University of Kentucky Markey Cancer Center, Lexington, KY 40536, USA; megan.hutchcraft@uky.edu (M.L.H.); fuela0@uky.edu (F.R.U.)

**Keywords:** OVA1, CA125, ovarian malignancy, early-stage detection

## Abstract

Background: Ovarian cancer is the deadliest gynecologic cancer, with no recommended screening test to assist with early detection. Cancer antigen 125 (CA125) is a serum biomarker commonly used by clinicians to assess preoperative cancer risk, but it underperforms in premenopausal women, early-stage malignancies, and several histologic subtypes. OVA1 is a multivariate index assay that combines CA125 and four other serum proteins to assess the malignant risk of an adnexal mass. Objective: To evaluate the performance of OVA1 in a cohort of patients with low-risk serum CA125 values. Study Design: We analyzed patient data from previous collections (N = 2305, prevalence = 4.5%) where CA125 levels were at or below 67 units/milliliter (U/mL) for pre-menopausal women and 35 U/mL for post-menopausal women. We compare the performance of OVA1 to CA125 in classifying the risk of malignancy in this cohort, including sensitivity, specificity, positive and negative predictive values. Results: The overall sensitivity of OVA1 in patients with a low-risk serum CA125 was 59% with a false-positive rate of 30%. OVA1 detected over 50% of ovarian malignancies in premenopausal women despite a low-risk serum CA125. OVA1 also correctly identified 63% of early-stage cancers missed by CA125. The most common epithelial ovarian cancer subtypes in the study population were mucinous (25%) and serous (23%) carcinomas. Despite a low-risk CA125, OVA1 successfully detected 83% of serous, 58% of mucinous, and 50% of clear cell ovarian cancers. Conclusions: As a standalone test, CA125 misses a significant number of ovarian malignancies that can be detected by OVA1. This is particularly important for premenopausal women and early-stage cancers, which have a much better long-term survival than late-stage malignancies. Using OVA1 in the setting of a normal serum CA125 can help identify at-risk ovarian tumors for referral to a gynecologic oncologist, potentially improving overall survival.

## 1. Introduction

Ovarian cancer is the deadliest gynecological cancer and the fifth leading cause of death in women. Although the incidence of ovarian cancer has declined in the past 30 years, the mortality rate remains high, with only half of women surviving longer than five years [1]. There is no recommended screening test for ovarian cancer, so women continue to present with advanced-stage disease where the prognosis is guarded [2]. Early detection of ovarian cancer is paramount if we hope to improve disease outcomes, including novel screening strategies and effective preoperative ovarian tumor evaluations.

Serum cancer antigen 125 (CA125) testing is only FDA-approved for monitoring women during the treatment of ovarian cancer. Still, many clinicians continue to use it as an off-label test to assess the risk of malignancy in women with an adnexal mass. While CA125 is an excellent marker for advanced-stage serous cancers, several ovarian cancer histological subtypes do not cause elevated CA125 levels, most notably mucinous carcinomas and non-epithelial malignancies [3]. Moreover, many early-stage ovarian cancers do not produce detectable levels of CA125 in the serum. For example, a 2014 analysis by Longoria et al. showed that CA125 identified only 69% of stage I primary ovarian malignancies using the CA125 cutoffs established by Dearking et al. (67 units/milliliter (U/mL) and 35 U/mL for premenopausal and postmenopausal women, respectively) [4,5].

The OVA1 assay is an FDA-approved multivariate index assay composed of five serum biomarkers, which collectively improve the detection of cancers missed by CA125 alone [6,7]. Each value is algorithmically composited into a unitless risk score between 1 and 10 to estimate the risk of malignancy of an adnexal mass that is scheduled for surgical removal [6,7]. Overall, the sensitivity of the multivariate OVA1 assay is 92% compared to 79% for CA125 testing alone [6]. In early-stage primary ovarian malignancies, the sensitivities are 91% and 73%, respectively [6]. In the pivotal OVA1 trial published in 2011, OVA1 identified 76% of malignancies missed by CA125 [7].

In this investigation, we evaluate the ability of the OVA1 multivariate assay to salvage detection of cancers in a cohort of patients in which CA125 is non-informative due to low-risk or “normal” serum values.

## 2. Materials and Methods

For this investigation, the authors reviewed clinical outcomes and serum biomarker data from five previous studies in the United States. These specimens were collected between 2009 and 2020 for clinical validation and independent verification of OVA1. The previous site collections were done according to five different protocols, including OVA1-001-CO1, OVA2-002-CO3, OVA1-7788, OVA1-PS1-CO4, and RP 01-2016. The purpose of each collection has previously been reported [6,7,8,9]. Each site independently obtained Institutional Review Board approval. Study inclusion criteria were the same at all locations, including women over 18 years undergoing surgical removal of an adnexal mass.

The authors merged the prospectively collected data for this retrospective analysis. Patients were eligible for inclusion in this study if the serum CA125 value was below the “high-risk” cutoff as established by Dearking et al. (67 U/mL premenopausal; 35 U/mL postmenopausal [5]). The analysis included tumors with malignant and benign pathology but did not include low malignant potential (borderline) tumors.

OVA1 is a multivariate index assay that incorporates transferrin, transthyretin (prealbumin), beta-2 microglobulin, apolipoprotein A-1, and CA125 (assays by Roche Diagnostics, Indianapolis, IN, USA). These markers were chosen to work in concert with proteins that were found to capture data patterns that would pick up malignancies missed by CA125 or reduce false-positives [10]. The algorithm combines the five biomarkers to generate a unitless risk score between 0 and 10 [6] (OvaCalc 3.0.3, Aspira Women’s Health, Austin, TX, USA). The risk of malignancy cutoffs are stratified as follows:

- Premenopausal women: ≥5.0 is elevated risk for malignancy
- Postmenopausal women: ≥4.4 is elevated risk for malignancy

Sensitivity, specificity, positive predictive value (PPV), and negative predictive value (NPV) were calculated for OVA1 and stratified by various subgroups. Statistics were calculated using the DTComPair package (version 1.0.3) of the R programming language (version 4.0.2, GNU GPL license).

## 3. Results

Of the 2305 patients with low-risk CA125 values, 104 were diagnosed with malignancy on final pathology (4.5% prevalence). Table 1 summarizes the clinical characteristics of the study population. Primary ovarian cancers accounted for 75 of the 104 malignancies (72%). The remaining 29 cancers included tumors metastatic to the ovary (9) and non-ovarian pelvic malignancies with no ovarian involvement (20). In patients diagnosed with cancer, OVA1 was high-risk in 61/104 (59% sensitivity; true-positive rate) and low-risk in 43 (41% false-negative rate). In patients with benign disease on final pathology, OVA1 was low-risk in 1548/2201 and high-risk in 653 (70% specificity, 97% NPV), with a false-positive rate of 30%.

Table 2 summarizes the influence of menopausal status on the performance of OVA1. For premenopausal women with a low-risk CA125 diagnosed with malignancy, the sensitivity and specificity of OVA1 were 51% and 77%, respectively. In postmenopausal women, OVA1 salvaged detection in 63% of the cancers which CA125 did not detect. Specificity was 61%, with PPV and NPV of 10% and 96%, respectively. In patients with benign disease on final pathology, OVA1 was low-risk in 980/1266 premenopausal women (77% specificity, 98% NPV) and 568/935 postmenopausal women (61% specificity, 96% NPV). The false-positive rate for premenopausal women was 23% and 39% for postmenopausal women.

Of the 75 primary ovarian cancers missed by CA125 testing alone, 58 had comprehensive staging. There were 41 early-stage (37 stage I; 4 stage II) and 17 advanced-stage cancers (11 stage 3; 6 stage 4). OVA1 was able to salvage detection in 63% (26/41) of early-stage cancers in which CA125 failed to detect malignancy, including 60% (22/37) of stage I and 100% (4/4) of stage 2 cancers (Table 2).

Since there is evidence in the literature to suggest that baseline CA125 levels may vary between racial groups [11,12,13,14], we examined the performance of OVA1 stratified by race (Table 3). Sixty-five white/Caucasian and twelve Black/African American women had cancer and a low-risk serum CA125. We excluded Hispanic/Latino and Asian populations from the individual race analysis because of the small sample size. For patients with a low-risk CA125, the sensitivity of OVA1 in white women was 65% versus 42% in Black women. Conversely, the specificity of OVA1 was higher in Black (79%) than in white women (68%). Positive and negative predictive values were similar. Likelihood ratios have also been provided. The positive likelihood ratio, LR+, shows the probability that an individual with a malignant adnexal mass will have an elevated risk OVA1 result compared to an individual with a benign mass. The negative likelihood ratio, LR−, shows the probability of an individual with a malignancy having a low-risk OVA1 result compared to one with a benign mass. For both Black and white women, these ratios were similar, though the LR− reflects the reduced sensitivity in Black women.

Table 4 shows the association between tumor histology and OVA1. When the CA125 was low-risk, OVA1 successfully detected serous carcinomas in 14/17 subjects (82%) and mucinous and clear cell cancers in 11/19 (58%) and 4/8 (50%), respectively. In addition to detecting epithelial ovarian cancer, OVA1 identified over half (5/8, 62%) of sex cord-stromal tumors and 40% (4/10) of granulosa cell tumors.

## 4. Discussion

The results of this investigation demonstrate that even if the CA125 is low-risk, OVA1 can effectively identify ovarian malignancies. OVA1 integrates CA125 with additional serum biomarkers to improve test sensitivity. High sensitivity helps OVA1 identify ovarian malignancies where CA125 frequently fails, like early-stage disease, premenopausal women, and several histologic subtypes, including mucinous, clear cell, and sex cord-stromal tumors (Table 1). Since serum CA125 alone is unreliable for determining the malignancy risk of an ovarian tumor [15,16,17,18,19,20], it is not recommended for use as a preoperative test.

The current guidelines published by the American College of Obstetricians and Gynecologists (ACOG) no longer recommend a specific CA125 cutoff value for use in premenopausal women [18]. Instead, the guidelines refer to a “very elevated” CA125 for premenopausal women, while the previously published ACOG recommendation was CA125 over 200 U/mL. Since CA125 is not approved for use as a preoperative test and ACOG recommends no specific premenopausal cutoff value, CA125 has a limited role in this cohort. When used as a preoperative test, a false-negative CA125 may adversely affect patient outcomes through missed cancer diagnoses, deferred referral to a cancer specialist, delayed surgical intervention, and repeat operations. This study evaluates the performance of OVA1 using a conservative CA125 cutoff of 67 U/mL [5], yet CA125 still fails to detect primary ovarian cancer in 39 premenopausal and 65 postmenopausal women. Conversely, OVA1 salvaged the detection of 51% (20/39) of premenopausal cancers and 63% (41/65) of postmenopausal cancers for which CA125 failed to detect malignancy.

The overall test sensitivity of OVA1 in detecting ovarian malignancy has previously been reported to exceed 90% [4,6,7], while serum CA125’s high sensitivity is limited to advanced-stage, high-grade serous ovarian cancers [19,20]. The advantage of a multivariate index assay like OVA1 is its ability to detect ovarian malignancies when CA125 is within the normal range. This is particularly critical for early-stage ovarian malignancies and premenopausal women, where appropriate treatment can result in favorable long-term outcomes. The five-year survival for stage I ovarian cancer exceeds 90% (75% for stage II disease) when treated by a gynecologic oncologist [21,22], compared to less than 50% for advanced-stage disease. However, since early-stage ovarian cancer is frequently asymptomatic, or symptoms are easily attributed to common conditions, diagnosis and referral are often delayed. In this study, OVA1 identified 63% (26/41) of early-stage primary ovarian malignancies missed by CA125. Similarly, Longoria et al. reported that OVA1 detected 78% of early-stage ovarian cancers missed by CA125 [4]. Therefore, a multivariate test like OVA1 may help clinicians identify more early-stage ovarian cancers for referral to a gynecologic oncologist, increasing the likelihood of proper treatment and improved long-term survival [23,24].

As a predictive and prognostic biomarker for ovarian cancer, sensitivity and specificity vary by histologic subtype [25]. Serum CA125 levels are frequently normal for ovarian malignancies of non-serous histology, especially for mucinous cancers where the true-positive rate is only 12% [25]. OVA1 identified at least half of clear cell (4/8, 50%) and mucinous (11/19, 58%) ovarian carcinomas missed by CA125, a promising result for these challenging cell types. Like serous cancers, early detection of mucinous and clear cell ovarian cancers confers a better prognosis, while the survival rate for late-stage cancers is guarded [26]. Half of all sex cord-stromal tumors (9/18) with a low-risk CA125 were also successfully identified by OVA1, including 7/12 early-stage malignancies.

This investigation has several strengths. The data were extracted from five prospective studies with similar inclusion criteria allowing for a large, homogenous study group with limited information bias. All ovarian tumor types were included in these studies, and most malignancies were appropriately staged. Biomarker testing was also independently performed and validated, limiting measurement error bias. A limitation of this study is the retrospective nature of the data analysis, which was performed after merging several study databases. Additionally, the percentage of early-stage ovarian cancer in this study (70%) is twice that expected in the general population, suggesting a possible sampling bias; however, this shift toward early-stage cancers does allow for a more robust evaluation of test performance in this cohort.

It is helpful to understand the impact of cancer prevalence on calculated predictive values. Study populations with a low cancer prevalence will have a lower PPV and higher NPV compared to a population of high prevalence. The OVA1 test performs with high sensitivity and NPV, but lower cancer specificity than CA125. Since OVA1 is intended as a first-line test for women with an adnexal mass that is planned for surgery, the test was specifically engineered to have high sensitivity and high NPV to avoid missing cancers (false-negative results). Missed cancers may require re-operation if an occult malignancy is found unexpectedly at surgery by a non-specialist, or cancers may present in a more advanced stage with potentially worse clinical outcomes. In this study population of low-risk CA125, OVA1 demonstrated a false-positive rate of 30%; however, it correctly identified 61/104 patients that were missed by CA125.

OVA1 is a sensitive multivariate biomarker test that can identify ovarian cancer even when the CA125 is normal. The findings of this investigation support previous publications [4,6,7] in concluding that OVA1 successfully identifies the majority of pelvic malignancies (59%) and 63% of early-stage ovarian cancers that are missed by serum CA125. Moreover, OVA1 identifies over half of the cancers in premenopausal women and non-serous ovarian malignancies when the CA125 is low-risk. OVA1 is a multivariate index assay with high sensitivity that can identify ovarian malignancy in the setting of a normal serum CA125 to help expedite surgical decisions and referral to a gynecologic oncologist, improving appropriate treatment and overall survival.

## Figures and Tables

**Table 1:** Summary of Clinical Characteristics for the Study Population *.

	All (N = 2305)		Pre-Menopausal (N = 1305)		Post-Menopausal ** (N = 1000)	
Race/Ethnicity						
	N	%	N	**%**	N	**%**
White/Caucasian	1492	64.73%	848	64.98%	644	64.40%
Black/African American	278	12.06%	192	14.71%	86	8.60%
Hispanic/Latinx	181	7.85%	128	9.81%	53	5.30%
Asian	34	1.48%	24	1.84%	10	1.00%
Native American	7	0.30%	4	0.31%	3	0.30%
Native Hawaiian/Pacific Islander	8	0.35%	6	0.46%	2	0.20%
Other	20	0.87%	13	1.00%	7	0.70%
Unspecified	285	12.36%	90	6.90%	195	19.50%
Pathology Diagnosis						
Benign	2201	95.49%	1266	97.01%	935	93.50%
Primary ovarian malignancy	75	3.25%	27	2.07%	48	4.80%
Non-primary, metastatic to ovary	9	0.39%	4	0.31%	5	0.50%
Non-primary, not metastatic to ovary	20	0.87%	8	0.61%	12	1.20%
Stage (Primary Ovarian Malignancies)						
Stage I	37	49.33%	16	59.26%	21	43.75%
Stage II	4	5.33%	1	3.70%	3	6.25%
Stage III	11	14.67%	2	7.41%	9	18.75%
Stage IV	6	8.00%	0	0.00%	6	12.50%
Not Staged	17	22.67%	8	29.63%	9	18.75%
Histological Subtype (Primary Ovarian Malignancies)						
Serous	17	22.67%	4	14.81%	13	27.08%
Mucinous	19	25.33%	8	29.63%	11	22.92%
Clear cell	8	10.67%	1	3.70%	7	14.58%
Endometrioid	3	4.00%	1	3.70%	2	4.17%
Carcinosarcoma	2	2.67%	0	0.00%	2	4.17%
Carcinoid	2	2.67%	1	3.70%	1	2.08%
Mixed	1	1.33%	0	0.00%	1	2.08%
Poorly differentiated	1	1.33%	0	0.00%	1	2.08%
Other epithelial cancer	1	1.33%	0	0.00%	1	2.08%
Germ cell	1	1.33%	1	3.70%	0	0.00%
Sex cord Stromal	8	10.67%	4	14.81%	4	8.33%
Granulosa cell tumor	10	13.33%	5	18.52%	5	10.42%
Other non-epithelial cancer	2	2.67%	2	7.41%	0	0.00%

* Low-risk CA125, 67 U/mL for premenopausa; 35 U/mL for postmenopausal or unknown. ** Includes patients with unspecified menopausal status.

**Table 2:** Clinical performance summary of OVA1 in low-risk CA125 patients.

	OVA1 Performance When CA125 Is Low-Risk *				
	N	Sensitivity (%, n/N)	Specificity (%, n/N)	PPV (%, n/N)	NPV (%, n/N)
All women	2305	58.65%	70.33%	8.54%	97.30%
		61/104	1548/2201	61/714	1548/1591
Pre-menopausal	1305	51.28%	77.41%	6.54%	98.10%
		20/39	980/1266	20/306	980/999
Post-menopausal or unknown	1000	63.08%	60.75%	10.05%	95.95%
		41/65	568/935	41/408	568/592
Early-stage (I and II)	41	63.41%			
		26/41			
Late-stage (III and IV)	17	76.47%			
		13/17			
Not staged	17	41.18%			
		7/17			

PPV, positive predictive value; NPV, negative predictive value. Cancer prevalence is 4.5% (104/2305). * Low-risk CA125, 67 U/mL for premenopausal subjects; 35 U/mL for postmenopausal or unknown subjects.

**Table 3:** OVA1 Performance Stratified by Race.

		OVA1 Performance When CA125 Is Low-Risk * by Race						
	Menopausal Status	N	Sensitivity (%, n/N)	Specificity (%, n/N)	PPV (%, n/N)	NPV (%, n/N)	LR+	LR−
White/Caucasian	All	1492	64.62%	67.90%	8.40%	97.68%	2.01	0.52
			42/65	969/1427	42/500	969/992		
	Pre	848	54.55%	75.18%	5.53%	98.42%	2.20	0.60
			12/22	621/826	12/217	621/631		
	Post	644	69.77%	57.90%	10.60%	96.40%	1.66	0.52
			30/43	348/601	30/283	348/361		
Black/African American	All	283	41.67%	79.32%	8.33%	96.79%	2.02	0.74
			5/12	211/266	5/60	211/218		
	Pre	195	37.50%	82.07%	8.33%	96.79%	2.09	0.76
			3/8	151/184	3/36	151/156		
	Post	88	50.00%	73.17%	8.33%	96.77%	1.86	0.68
			2/4	60/82	2/24	60/62		

PPV, positive predictive value; NPV, negative predictive value; LR+, positive likelihood ratio; LR−, negative likelihood ratio. Cancer prevalence is 4.5% (104/2305). * Low-risk CA125, 67 U/mL for premenopausal subjects; 35 U/mL for postmenopausal or unknown subjects.

**Table 4:** OVA1 Performance by Histological Subtype and FIGO Stage When Low-Risk CA125 *.

	OVA1 n/N					
Histological Subtype	All	Stage I	Stage II	Stage III	Stage IV	Not Staged
Serous	14/17	2/2	2/2	5/5	4/5	1/3
Mucinous	11/19	7/13		2/3		2/3
Clear cell	4/8	3/4		0/2		1/2
Endometrioid	1/3	1/2				0/1
Carcinosarcoma	2/2		1/1	1/1		
Carcinoid	0/2	0/1				0/1
Mixed	1/1					1/1
Poorly differentiated	1/1				1/1	
Other epithelial cancer	1/1	1/1				
Germ cell	1/1	1/1				
Sex cord stromal	5/8	3/5	1/1			1/2
Granulosa cell tumor	4/10	3/6				1/4
Other non-epithelial cancer	1/2	1/2				

* Low-risk CA125, 67 U/mL for premenopausal subjects; 35 U/mL for postmenopausal or unknown subjects.

## Data Availability

All data analyzed in this study is proprietary.
